# The Potential of Dehydrated *Geniotrigona thoracica* Stingless Bee Honey against Metabolic Syndrome in Rats Induced by a High-Carbohydrate, High-Fat Diet

**DOI:** 10.3390/ph17111427

**Published:** 2024-10-24

**Authors:** Liyana Nabihah Ikhsan, Kok-Yong Chin, Fairus Ahmad

**Affiliations:** 1 Department of Anatomy, Faculty of Medicine, Universiti Kebangsaan Malaysia, Jalan Yaacob Latif, Bandar Tun Razak, Kuala Lumpur 56000, Malaysia; 2 Department of Pharmacology, Faculty of Medicine, Universiti Kebangsaan Malaysia, Jalan Yaacob Latif, Bandar Tun Razak, Kuala Lumpur 56000, Malaysia

**Keywords:** honey, stingless bee honey, honey dehydration, anti-inflammatory, obesity, *Geniotrigona thoracica*, HCHF diet

## Abstract

Background/Objectives: Metabolic syndrome (MS) is diagnosed when at least three out of five key risk factors are present: obesity, high blood pressure, insulin resistance, high triglycerides (TG) and low high-density lipoprotein (HDL). MS is often associated with chronic low-grade inflammation. Recent studies have shown that raw stingless bee honey (SBH) can alleviate MS risk factors. However, the high moisture content in raw SBH predisposes it to fermentation, which can degrade its quality. Therefore, dehydrating SBH is necessary to prevent the fermentation process. This study aimed to compare the effects of dehydrated (DeGT) and raw (RGT) SBH from *Geniotrigona thoracica* species on high-carbohydrate, high-fat diet (HCHF)-induced MS in rats. Methods: Twenty-four male Wistar rats were divided into four groups: control (C), HCHF-induced MS without treatment (MS), HCHF-induced MS treated with DeGT (MS+DeGT) and HCHF-induced MS treated with RGT (MS+RGT). Group C received standard rat chow, while the other groups were fed with HCHF diet for 16 weeks. In the final eight weeks, two HCHF-induced groups received their respective SBH treatments. Results: Both DeGT and RGT treatments reduced energy intake, fat mass, high blood pressure, inflammatory (tumour necrosis factor-alpha (TNF-α)) and obesity (the leptin/adiponectin (L/A) ratio, corticosterone, 11 beta-hydroxysteroid dehydrogenase type-1 (11βHSD1)) markers, as well as prevented histomorphometry changes (prevented adipocyte hypertrophy, increased the Bowman’s space area and glomerular atrophy). Additionally, DeGT increased serum HDL levels, while RGT reduced serum TG, leptin and other inflammatory markers (interleukin-6 (IL-6) and interleukin-1 beta (IL-1β)), as well as hepatosteatosis. Conclusions: While DeGT demonstrates potential as a preventive agent for MS, RGT exhibited more pronounced anti-MS effects in this study.

## 1. Introduction

Metabolic syndrome (MS) is a metabolic imbalance characterized by several risk factors, including obesity, hypertension, insulin resistance and dyslipidemia. Between 2011 and 2018, approximately 35–38% of adults in the United States were diagnosed with MS [1]. In the Asia Pacific region, the prevalence of MS ranges from 11.9–37.1% among adults [2]. MS has now emerged as a worldwide public health concern due to its association with a five-fold increase in the risk of developing type II diabetes mellitus (T2DM) and a three-fold increase in developing cardiovascular diseases [3,4]. The prevalence of diabetes among individuals aged 20–79 globally was 8.8% in 2015 and is expected to reach 10.8% in 2040 [5].

The rise in MS is attributed to modern lifestyle changes. Two prominent factors contributing to this rise are the consumption of high-calorie, low-fiber fast food and a reduction in physical activity due to sedentary lifestyles [6]. The primary approach to managing MS involves adopting a healthy lifestyle, including a balanced diet, calorie control and regular physical activity. However, pharmacological or surgical interventions are necessary to manage MS risk factors effectively [7]. Pharmacological treatment often involves a combination of drugs, as no single medication can simultaneously treat all MS risk factors [8]. Consequently, this situation can lead to polypharmacy, which increases the risk of drug interactions and side effects as well as hinders patient’s compliance with medication regimens [9].

In recent years, there has been growing interest in exploring the potential benefits of natural products in the treatment of MS, including green tea, cinnamon, cocoa, coffee and honey [10,11]. Honey, renowned for its various biological activities, holds promising prospects in preventing MS [12]. A study on the types of honey in Malaysia revealed that stingless bee honey (SBH) remains relatively underexplored [13]. In addition possessing high antioxidants, owing to its polyphenol [14], SBH is also rich in probiotics [15]. Unlike other honey which predominantly composed of fructose and glucose, SBH is unique because it contains trehalulose, a natural sucrose isomer as the main sugar [16,17]. Trehalulose has a low glycemic index; hence, SBH has the potential as an anti-diabetic agent [16]. Research has shown trehalose treatment on obese mice can prevent insulin resistance and adipocyte hypertrophy [18,19]. Among the various species of SBH worldwide, the most commonly domesticated species in Malaysia are the *Heterotrigona itama* (*H. itama*) and *Geniotrigona thoracica* (*G. thoracica*) species [20]. SBH had shown promising therapeutic potential in the treatment of MS. SBH from *H. itama* species can prevent obesity, hypertension and dyslipidemia [21,22]. Meanwhile, SBH from *G. thoracica* species can prevent hyperglycemia and dyslipidemia [23].

However, SBH has storage problems because of its high moisture content. Moisture content in honey is a crucial parameter because excess moisture promotes microbial contamination and fermentation process, thus shortening the lifespan of honey [24]. Fermentation also makes SBH more acidic [25]. Previous studies have shown that SBH has higher moisture and acidity than other honey types [26,27]. The dehydration process is necessary for SBH to reduce its moisture content, protecting it from microorganism contamination [25,28]. Subsequently, this can preserve the stability and beneficial properties of SBH [24]. Therefore, the fermentation process and undesirable increase in SBH acidity can be prevented, making it more palatable. However, there is a lack of studies to understand whether the dehydration process will affect the biological activities of honey.

To date, there have been no studies exploring the potential of dehydrated SBH in the treatment of MS. Therefore, this study aims to compare the potential benefits of dehydrated SBH (DeGT) with raw SBH (RGT) from *G. thoracica* species in a rat model of high-carbohydrate, high-fat diet (HCHF)-induced MS. This is to determine whether the dehydrated form of SBH exerts similar, superior or reduced effects on MS rats compared to raw SBH. If dehydrated SBH is as effective as raw SBH in preventing MS, the SBH can be dehydrated after harvesting. This will overcome storage challenges and preserve honey quality.

## 2. Results

### 2.1. Honey Analysis

#### 2.1.1. Physicochemical Properties Analysis

Table 1 shows the physicochemical properties of DeGT and RGT. The dehydration process reduced the moisture content from 31% to 14.3%. This reduction was accompanied by a decrease in acidity, indicated by an increase in pH from 3.3 to 3.5 and a decrease in free acidity from 274.8 to 138 mEq/kg. The hydroxymethylfurfural (HMF) content was reduced from 0.3 to <0.1 mg/kg. The ash content and diastase activity remained unchanged at 0.2 g/100 g and <1 DN, respectively. For sugar content, the trehalulose, fructose and glucose level slightly decreased from 52 to 48.5, from 8.5 to 8.1 and from 11 to 7.3 g/100 g, respectively. The content of sucrose and maltose remained the same, both of which were <0.01 g/100 g.

#### 2.1.2. Liquid Chromatography–Mass Spectrometry (LC-MS) Analysis

Table 2 shows the bioactive compounds in DeGT and RGT. DeGT contained 33 bioactive compounds comprising 10 types of sugars, two amino acids, two phenolics, four flavonoids, five iridoid glycosides, one phenylpropanoid and nine other organic compounds. Meanwhile, RGT contained 25 bioactive compounds comprising six types of sugars, two amino acids, three phenolics, four flavonoids, three iridoid glycosides, one phenylpropanoid and six other organic compounds.

### 2.2. Energy Intake

**Figure 1:**
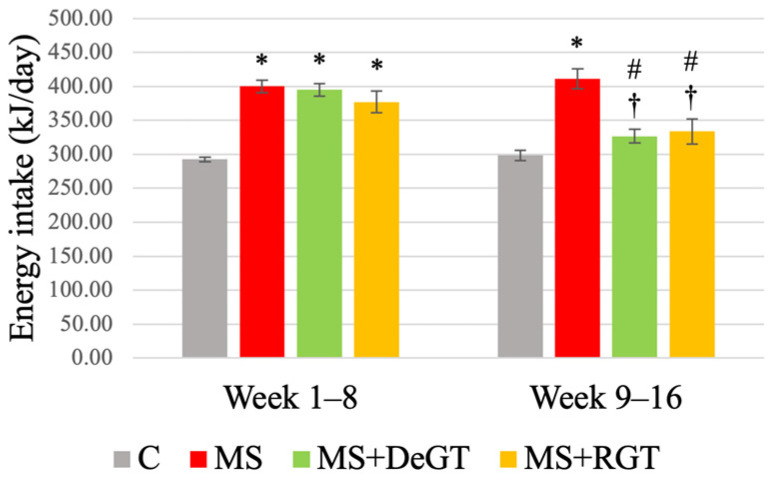
Energy intake at week 1–8 and week 9–16. All data are presented as mean ± SEM. Symbol ‘*’ indicates a significant difference compared to the C group in the same week (*p* < 0.05), ‘#’ indicates a significant difference compared to the MS group in the same week (*p* < 0.05) and ‘†’ indicates a significant difference at week 16 compared to week 8 (*p* < 0.05). Abbreviations: C (control), MS (HCHF diet-induced rats without treatment), MS+DeGT (HCHF diet-induced rats treated with dehydrated SBH from *G. thoracica* species) and MS+RGT (HCHF diet-induced rats treated with raw SBH from *G. thoracica* species).

From week 1 to week 8, the HCHF-induced rats (MS, MS+DeGT and MS+RGT) showed a significant increase in daily energy intake compared to the C group (*, *p* < 0.05) (Figure 1). From week 9 to week 16, both DeGT and RGT treatments showed a significant reduction in daily energy intake compared to the MS group and week 1–8 (# and †, *p* < 0.05).

### 2.3. Anthropometric Measurements (Body Weight, Body Mass Index (BMI) and Abdominal Circumference)

**Figure 2:**
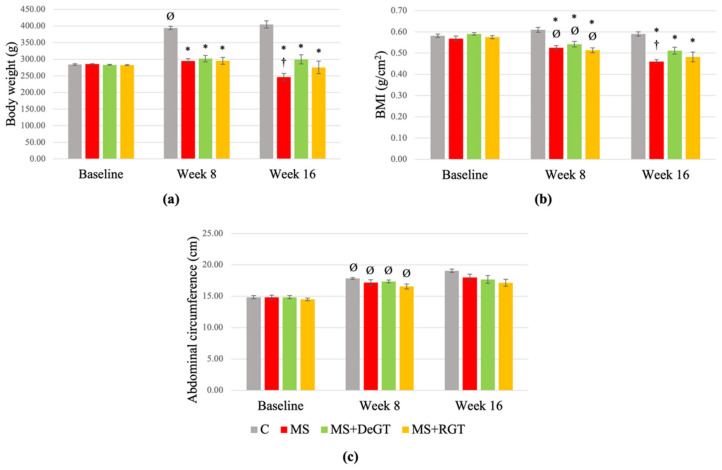
Anthropometric measurements comprise of (**a**) body weight, (**b**) body mass index (BMI) and (**c**) abdominal circumference at baseline, week 8 and week 16. All data are presented as mean ± SEM. Symbol ‘*’ indicates a significant difference compared to the C group in the same week (*p* < 0.05), ‘Ø’ indicates a significant difference in the same group at week 8 compared to baseline (*p* < 0.05) and ‘†’ indicates a significant difference in the same group at week 16 compared to week 8 (*p* < 0.05). Abbreviations: C (control), MS (HCHF diet-induced rats without treatment), MS+DeGT (HCHF diet-induced rats treated with dehydrated SBH from *G. thoracica* species) and MS+RGT (HCHF diet-induced rats treated with raw SBH from *G. thoracica* species).

Body weights of HCHF-induced rats (MS, MS+DeGT and MS+RGT) were significantly lower compared to the C group at week 8 (*, *p* < 0.05), while the body weights of the C group significantly increased at week 8 compared to the baseline (Ø, *p* < 0.05) (Figure 2a). Meanwhile, BMIs of the HCHF-induced rats (MS, MS+DeGT and MS+RGT) at week 8 were significantly lower compared to the C group and baseline (* and Ø, *p* < 0.05) (Figure 2b). Although there was a significant increase in abdominal circumference in all the groups at week 8 compared to the baseline (Ø, *p* < 0.05), there was no significant difference between all the groups at week 8 (*p* > 0.05) (Figure 2c).

At week 16, body weights and BMIs of the MS, MS+DeGT and MS+RGT groups were significantly lower compared to the C group (*, *p* < 0.05), and both treatments showed no significant changes in those parameters compared to the MS group (*p* > 0.05) (Figure 2a,b). Meanwhile, the MS group showed a significant decrease in body weight and BMI at week 16 compared to week 8 (†, *p* < 0.05) (Figure 2a,b). Otherwise, both DeGT and RGT treatments showed no significant changes in abdominal circumference at week 16 compared to the MS group or week 8 (*p* > 0.05) (Figure 2c).

### 2.4. Body Composition Based on Dual-Energy X-ray Absorptiometry (DXA) Scan (Fat Mass and Lean Mass)

**Figure 3:**
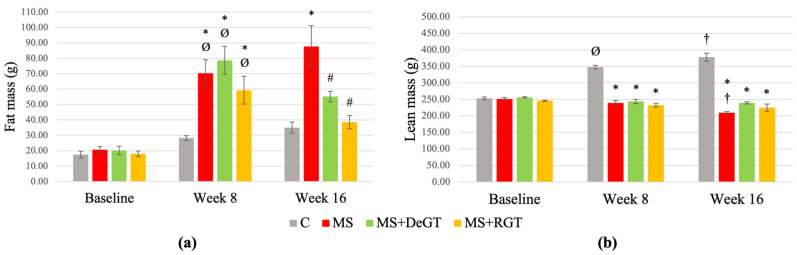
Body composition based on Dual-Energy X-Ray Absorptiometry (DXA) scan, including (**a**) fat mass and (**b**) lean mass at baseline, week 8 and week 16. All data are presented as mean ± SEM. Symbol ‘*’ indicates a significant difference compared to the C group in the same week (*p* < 0.05), ‘#’ indicates a significant difference compared to the MS group in the same week (*p* < 0.05), ‘Ø’ indicates a significant difference in the same group at week 8 compared to baseline (*p* < 0.05) and ‘†’ indicates a significant difference in the same group at week 16 compared to week 8 (*p* < 0.05). Abbreviations: C (control), MS (HCHF diet-induced rats without treatment), MS+DeGT (HCHF diet-induced rats treated with dehydrated SBH from *G. thoracica* species) and MS+RGT (HCHF diet-induced rats treated with raw SBH from *G. thoracica* species).

At week 8, HCHF-induced rats (MS, MS+DeGT and MS+RGT) showed a significant increase in fat mass compared to the C group and baseline (* and Ø, *p* < 0.05) (Figure 3a). The lean masses of HCHF-induced rats (MS, MS+DeGT and MS+RGT) were significantly lower compared to the C group at week 8 (*, *p* < 0.05), while the lean masses of the C group significantly increased at week 8 compared to the baseline (Ø, *p* < 0.05) (Figure 3b).

At week 16, both DeGT and RGT treatments showed a significant decrease in fat mass compared to the MS group (#, *p* < 0.05) (Figure 3a). Lean masses of the MS, MS+DeGT and MS+RGT groups were significantly lower compared to the C group at week 16 (*, *p* < 0.05), and both treatments showed no significant changes compared to the MS group at week 16 (*p* > 0.05) (Figure 3b). Meanwhile, the MS group showed a significant decrease in lean mass at week 16 compared to week 8 (†, *p* < 0.05) (Figure 3b).

### 2.5. Systolic and Diastolic Blood Pressure

**Figure 4:**
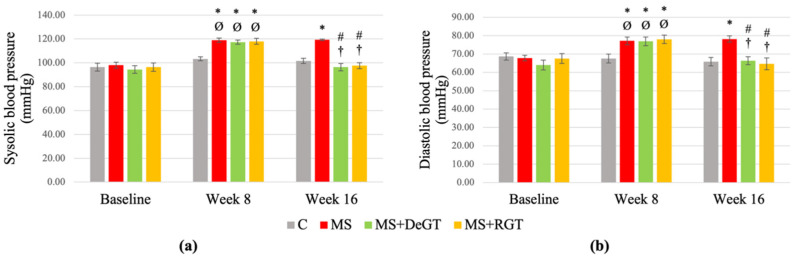
Blood pressure, including (**a**) systolic and (**b**) diastolic blood pressure at baseline, week 8 and week 16. All data are presented as mean ± SEM. Symbol ‘*’ indicates a significant difference compared to the C group in the same week (*p* < 0.05), ‘#’ indicates a significant difference compared to the MS group in the same week (*p* < 0.05), ‘Ø’ indicates a significant difference in the same group at week 8 compared to baseline (*p* < 0.05) and ‘†’ indicates a significant difference in the same group at week 16 compared to week 8 (*p* < 0.05). Abbreviations: C (control), MS (HCHF diet-induced rats without treatment), MS+DeGT (HCHF diet-induced rats treated with dehydrated SBH from *G. thoracica* species) and MS+RGT (HCHF diet-induced rats treated with raw SBH from *G. thoracica* species).

At week 8, the HCHF-induced rats (MS, MS+DeGT and MS+RGT) showed a significant increase in systolic and diastolic blood pressure compared to the C group and baseline (* and Ø, *p* < 0.05) (Figure 4a,b). At week 16, both the DeGT and RGT treatments reduced systolic and diastolic blood pressure significantly compared to the MS group and week 8 (# and †, *p* < 0.05).

### 2.6. Insulin Resistance

**Figure 5:**
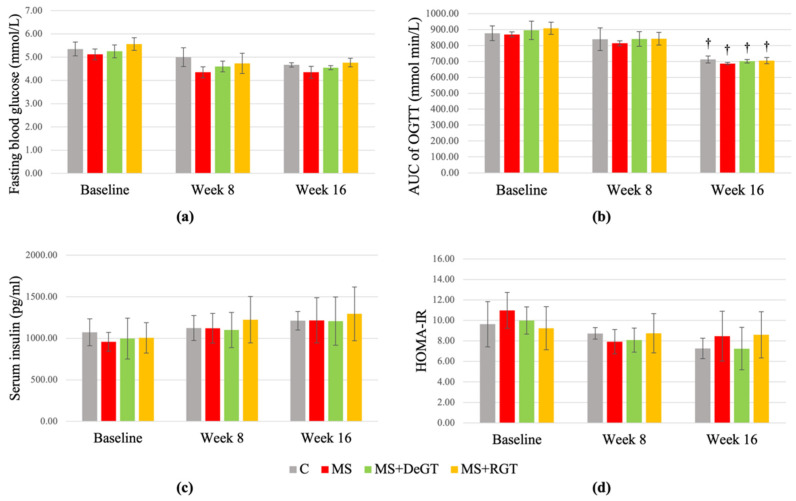
Insulin resistance measurements, including (**a**) fasting lipid profile, (**b**) area under the cure of oral glucose tolerance test (AUC of OGTT), (**c**) serum insulin and (**d**) Homeostatic Model Assessment for Insulin Resistance (HOMA-IR) at baseline, week 8 and week 16. All data are presented as mean ± SEM. Symbol ‘†’ indicates a significant difference in the same group at week 16 compared to week 8 (*p* < 0.05). Abbreviations: C (control), MS (HCHF diet-induced rats without treatment), MS+DeGT (HCHF diet-induced rats treated with dehydrated SBH from *G. thoracica* species) and MS+RGT (HCHF diet-induced rats treated with raw SBH from *G. thoracica* species).

At week 8, fasting blood glucose, area under the curve of oral glucose tolerance test (AUC of OGTT), serum insulin and homeostatic model assessment for insulin resistance (HOMA-IR) of the HCHF-induced rats (MS, MS+DeGT and MS+RGT) showed no significant difference compared to the C group or baseline (*p* > 0.05) (Figure 5a–d). At week 16, both DeGT and RGT treatments showed no significant changes in fasting blood glucose, serum insulin and HOMA-IR compared to the MS group or week 8 (*p* > 0.05) (Figure 5a,c,d). Although there was a significant decrease in AUC of OGTT in all the groups at week 16 compared to week 8 (†, *p* < 0.05), there was no significant difference between all the groups at week 16 (*p* > 0.05) (Figure 5b).

### 2.7. Serum Fasting Lipid Profile

**Figure 6:**
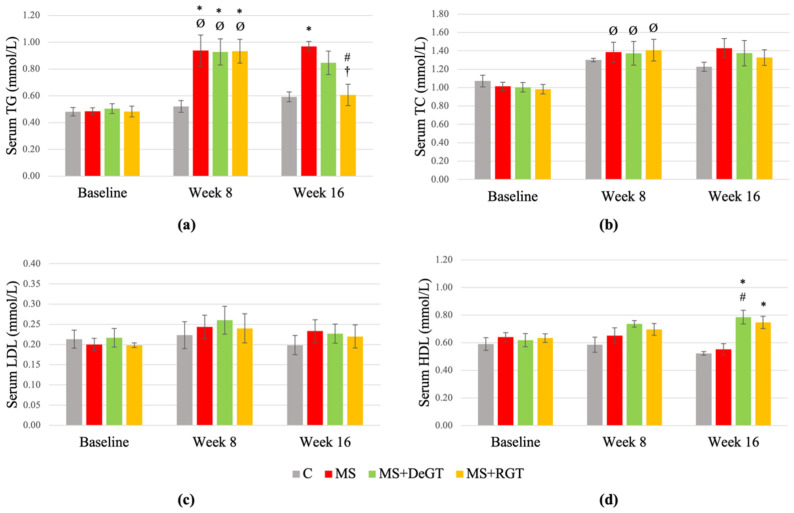
Serum fasting lipid profile, including (**a**) triglycerides (TG), (**b**) total cholesterol (TC), (**c**) low-density lipoprotein (LDL) and (**d**) high-density lipoprotein (HDL) at baseline, week 8 and week 16. All data are presented as mean ± SEM. Symbol ‘*’ indicates a significant difference compared to the C group in the same week (*p* < 0.05), ‘#’ indicates a significant difference compared to the MS group in the same week (*p* < 0.05), ‘Ø’ indicates a significant difference in the same group at week 8 compared to baseline (*p* < 0.05) and ‘†’ indicates a significant difference in the same group at week 16 compared to week 8 (*p* < 0.05). Abbreviations: C (control), MS (HCHF diet-induced rats without treatment), MS+DeGT (HCHF diet-induced rats treated with dehydrated SBH from *G. thoracica* species) and MS+RGT (HCHF diet-induced rats treated with raw SBH from *G. thoracica* species).

At week 8, the HCHF-induced rats (MS, MS+DeGT and MS+RGT) showed a significant increase in serum triglycerides (TG) compared to the C group and baseline (* and Ø, *p* < 0.05) (Figure 6a). Although there was a significant increase in serum total cholesterol (TC) of the HCHF-induced rats (MS, MS+DeGT and MS+RGT) at week 8 compared to the baseline (Ø, *p* < 0.05), there was no significant difference compared to the C group at week 8 (*p* > 0.05) (Figure 6b). Otherwise, there were no significant differences in the serum low-density lipoprotein (LDL) and high-density lipoprotein (HDL) of HCHF-induced rats (MS, MS+DeGT and MS+RGT) at week 8 compared to the C group or baseline (*p* > 0.05) (Figure 6c,d).

At week 16, only RGT treatment significantly decreased serum TG compared to the MS group and week 8 (# and †, *p* < 0.05) (Figure 6a). Although both DeGT and RGT treatments showed a significant increase in serum HDL compared to the C group (*, *p* < 0.05), only DeGT treatment significantly increased serum HDL compared to the MS group (#, *p* < 0.05) (Figure 6d). Otherwise, both DeGT and RGT treatments showed no significant changes in serum TC and LDL at week 16 compared to the MS group or week 8 (*p* > 0.05) (Figure 6b,c).

### 2.8. Inflammatory Markers in Adipose Tissue

**Figure 7:**
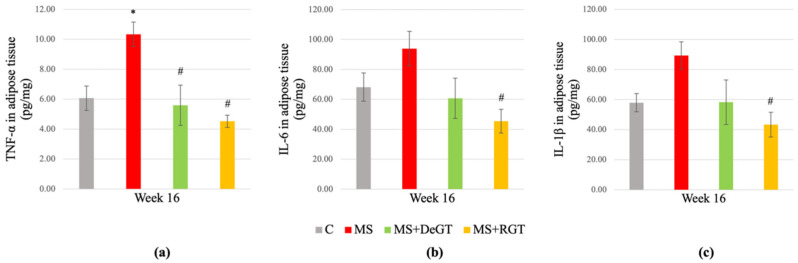
Inflammatory markers in adipose tissue at the end of the study consisting of (**a**) tumor necrosis factor-alpha (TNF-α), (**b**) interleukin-6 (IL-6) and (**c**) interleukin-1 beta (IL-1β). All data are presented as mean ± SEM. Symbol ‘*’ indicates a significant difference compared to the C group (*p* < 0.05) and ‘#’ indicates a significant difference compared to the MS group (*p* < 0.05). Abbreviations: C (control), MS (HCHF diet-induced rats without treatment), MS+DeGT (HCHF diet-induced rats treated with dehydrated SBH from *G. thoracica* species) and MS+RGT (HCHF diet-induced rats treated with raw SBH from *G. thoracica* species).

At week 16, this study showed a significant increase in tumor necrosis factor-alpha (TNF-α) (*, *p* < 0.05), while no significant changes were observed in interleukin-6 (IL-6) and interleukin-1 beta (IL-1β) (*p* > 0.05) in the adipose tissue of the MS group compared to the C group (Figure 7a–c). Both DeGT and RGT treatments significantly reduced TNF-α levels in adipose tissue compared to the MS group (#, *p* < 0.05) (Figure 7a). However, only RGT treatment showed a significant reduction in IL-6 and IL-1β levels in adipose tissue compared to the MS group (#, *p* < 0.05) (Figure 7b,c).

### 2.9. Obesity Markers in Serum and Adipose Tissue

**Figure 8:**
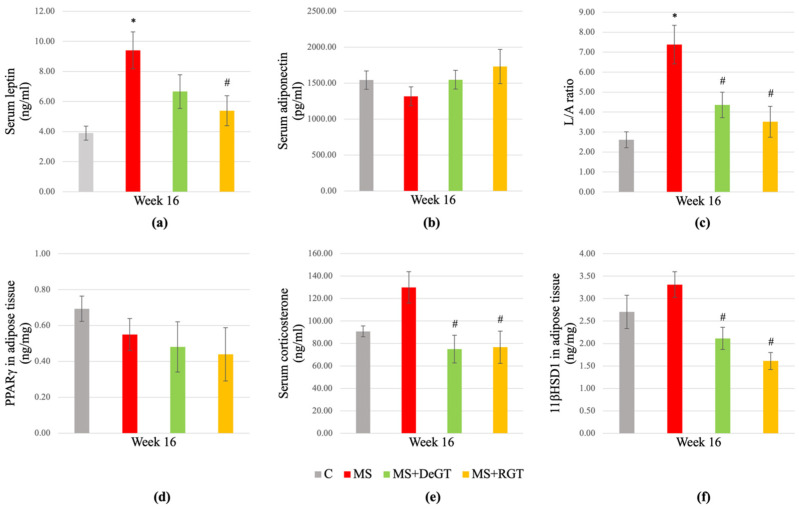
Obesity markers at the end of the study consisting of (**a**) serum leptin, (**b**) serum adiponectin, (**c**) leptin/adiponectin (L/A) ratio, (**d**) peroxisome proliferator-activated receptor γ (PPARγ) in adipose tissue (**e**) serum corticosterone and (**f**) 11 beta-hydroxysteroid dehydrogenase type-1 (11βHSD1) in adipose tissue. All data are presented as mean ± SEM. Symbol ‘*’ indicates a significant difference compared to the C group (*p* < 0.05) and ‘#’ indicates a significant difference compared to the MS group (*p* < 0.05). Abbreviations: C (control), MS (HCHF diet-induced rats without treatment), MS+DeGT (HCHF diet-induced rats treated with dehydrated SBH from *G. thoracica* species) and MS+RGT (HCHF diet-induced rats treated with raw SBH from *G. thoracica* species).

At week 16, serum leptin and leptin/adiponectin (L/A) ratio increased significantly in the MS group compared to the C group (*, *p* < 0.05) (Figure 8a,c). Meanwhile, there were no significant differences in serum adiponectin, serum corticosterone, as well as peroxisome proliferator-activated receptor γ (PPARγ) and 11 beta-hydroxysteroid dehydrogenase type-1 (11βHSD1) levels in adipose tissue of the MS group compared to the C group (*p* > 0.05) (Figure 8b,d–f).

Both DeGT and RGT treatments significantly reduced serum L/A ratio, serum corticosterone and 11βHSD1 level in adipose tissue compared to the MS group at week 16 (#, *p* < 0.05) (Figure 8c,e,f). However, only RGT treatment significantly reduced serum leptin compared to the MS group at week 16 (#, *p* < 0.05) (Figure 8a). Meanwhile, both treatments showed no significant changes in serum adiponectin and PPARγ levels in adipose tissue compared to the MS group at week 16 (*p* > 0.05) (Figure 8b,d).

### 2.10. Histomorphometry of Adipose, Liver and Kidney Tissue

#### 2.10.1. Adipose Tissue

**Figure 9:**
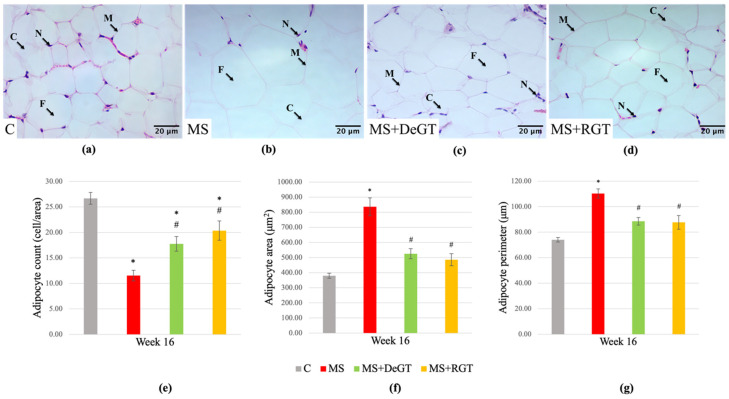
Photomicrograph of visceral adipose tissue in (**a**) C, (**b**) MS, (**c**) MS+DeGT and (**d**) MS+RGT rats under a light microscope at 400× magnification. Abbreviations: N (nucleus), C (cytoplasm), M (plasma membrane) and F (fat vacuole). The size of adipocytes is indicated by the measurement of (**e**) adipocyte cell count per area, (**f**) adipocyte area and (**g**) adipocyte perimeter. All data are presented as mean ± SEM. Symbol ‘*’ indicates a significant difference compared to the C group (*p* < 0.05) and ‘#’ indicates a significant difference compared to the MS group (*p* < 0.05). Abbreviations: C (control), MS (HCHF diet-induced rats without treatment), MS+DeGT (HCHF diet-induced rats treated with dehydrated SBH from *G. thoracica* species) and MS+RGT (HCHF diet-induced rats treated with raw SBH from *G. thoracica* species).

At the end of the study, the MS group showed adipocyte hypertrophy, as evidenced by a significant reduction in adipocyte cell count per area as well as a significant increase in adipocyte area and perimeter compared to the C group (*, *p* < 0.05) (Figure 9a,b,e–g). Both DeGT and RGT treatments prevented adipocyte hypertrophy, as indicated by a significant increase in adipocyte cell count per area as well as a significant reduction in adipocyte area and perimeter compared to the MS group at week 16 (#, *p* < 0.05) (Figure 9b–g).

#### 2.10.2. Liver Tissue

**Figure 10:**
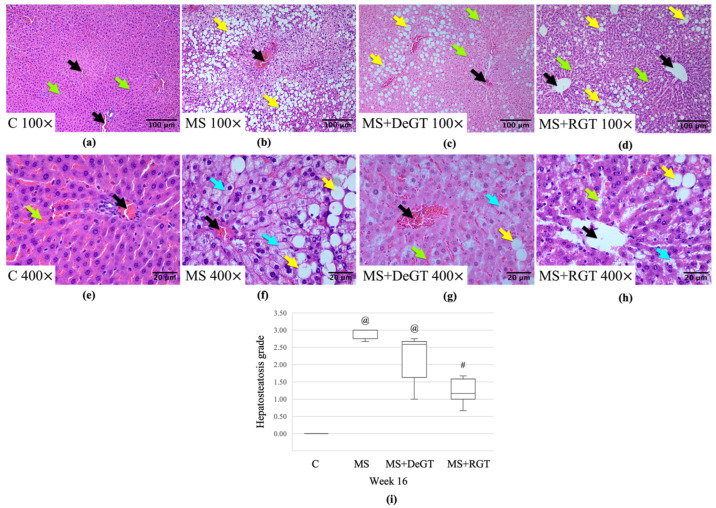
Photomicrograph of liver tissue in (**a**,**e**) C, (**b**,**f**) MS, (**c**,**g**) MS+DeGT and (**d**,**h**) MS+RGT rats under a light microscope at 100× and 400× magnification, respectively. Abbreviations: central vein (black arrow), sinusoids (green arrow), macrosteatosis (yellow arrow) and microsteatosis (blue arrow). The hepatosteatosis is confirmed by the measurement of (**i**) hepatosteatosis grade. The data are presented as median (IQR). Symbol ‘@’ indicates a significant difference compared to the C group (*p* < 0.05) and ‘#’ indicates a significant difference compared to the MS group (*p* < 0.05). Abbreviations: C (control), MS (HCHF diet-induced rats without treatment), MS+DeGT (HCHF diet-induced rats treated with dehydrated SBH from *G. thoracica* species) and MS+RGT (HCHF diet-induced rats treated with raw SBH from *G. thoracica* species).

MS and MS+DeGT group showed higher hepatic macrosteatosis and microsteatosis, as confirmed by significantly higher hepatosteatosis grade compared to the C group at week 16 (@, *p* < 0.05) (Figure 10a–c,e–g,i). Meanwhile, only RGT treatment reduced hepatic macrosteatosis and microsteatosis, as indicated by a significant reduction in hepatosteatosis grade compared to the MS group (#, *p* < 0.05) (Figure 10b,d,f,h,i).

#### 2.10.3. Kidney Tissue

**Figure 11:**
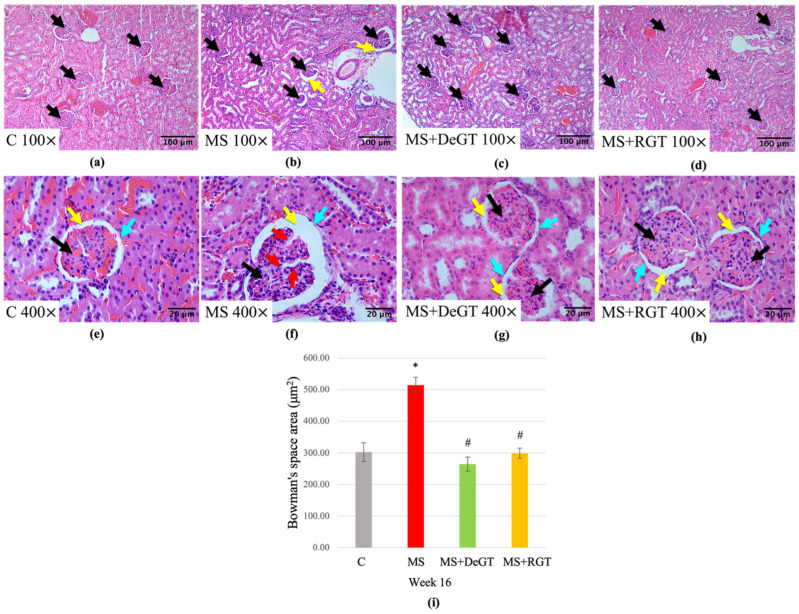
Photomicrograph of kidney tissue in (**a**,**e**) C, (**b**,**f**) MS, (**c**,**g**) MS+DeGT and (**d**,**h**) MS+RGT rats under a light microscope at 100× and 400× magnification, respectively. Abbreviations: glomerulus (black arrow), Bowman’s space (yellow arrow), renal corpuscle (blue arrow) and glomerular atrophy (red arrow). The enlargement of Bowman’s space is indicated by the measurement of (**i**) Bowman’s space area. The data are presented as mean ± SEM. Symbol ‘*’ indicates a significant difference compared to the C group (*p* < 0.05) and ‘#’ indicates a significant difference compared to the MS group (*p* < 0.05). Abbreviations: C (control), MS (HCHF diet-induced rats without treatment), MS+DeGT (HCHF diet-induced rats treated with dehydrated SBH from *G. thoracica* species) and MS+RGT (HCHF diet-induced rats treated with raw SBH from *G. thoracica* species).

At week 16, the MS group showed a significant increase in Bowman’s space area compared to the C group (*, *p* < 0.05) (Figure 11a,b,e,f,i). Histological observation at 400× magnification showed glomerular atrophy in the MS group compared to the C group (Figure 11e,f). Both DeGT and RGT treatments prevented the increase in Bowman’s space area compared to the MS group at week 16 (#, *p* < 0.05) (Figure 11b–d,f–i). Based on 400× magnification histological observation, both treatments prevented glomerular atrophy compared to the MS group (Figure 11f–h).

## 3. Discussion

The present study compared the effect of DeGT and RGT treatments on HCHF-induced MS rats. During the initial 8 weeks of HCHF induction, the rats exhibited increased energy intake, fat mass, blood pressure and serum TG. At week 16, there were increases in inflammatory (TNF-α in adipose tissue) and obesity (serum leptin, serum L/A ratio) markers. Additionally, the histomorphometry changes (adipocyte hypertrophy, hepatosteatosis, increase in Bowman’s space area and glomerular atrophy) were observed. This study demonstrated that both DeGT and RGT treatments for 8 weeks effectively reduced energy intake, fat mass, high blood pressure, inflammatory (TNF-α in adipose tissue) and obesity (serum L/A ratio, serum corticosterone, 11βHSD1 in adipose tissue) markers, as well as histomorphometry changes (adipocyte hypertrophy, Bowman’s space area and glomerular atrophy). Although DeGT increased serum HDL, DeGT could not reduce serum TG, IL-6 and IL-1β adipose tissue, serum leptin and hepatosteatosis in contrast to the effects observed in RGT treatment.

In this study, the HCHF diet for 8 and 16 weeks successfully induced MS by inducing at least three out of five risk factors: obesity, hypertension and hypertriglyceridemia, which are aligned with previous studies [21,22]. High-calorie intake can lead to the accumulation of excess calories in the form of body fat [29]. Excess adipose tissue oversecretes leptin, which can cause leptin resistance [30]. Leptin resistance hinders the optimal functioning of the leptin hormone in regulating satiety [31]. In this study, energy intake increased in HCHF-induced rats, which was consistent with elevated serum leptin levels. Therefore, it is hypothesized that the high energy intake in HCHF-induced rats in the study may be attributed to the development of leptin resistance.

In this study, the HCHF diet induced obesity as evidenced by an increase in fat mass. Histomorphometry results further confirmed the presence of adipocyte hypertrophy indicated by a reduction in adipocyte cell count per area, and a significant increase in adipocyte area and perimeter. Additionally, excess fat deposition in ectopic tissue was observed in the liver and confirmed by an increase in hepatosteatosis grade. However, the findings of this study showed no significant increase in anthropometric parameters: body weight, BMI and abdominal circumference in HCHF-induced rats. The lack of observable results may be attributed to the fact that the anthropometric parameters serve as a surrogate measurement for obesity and do not measure visceral fat directly [32,33]. Nevertheless, our findings suggest that the HCHF diet employed in this study can induce obesity, despite no apparent alterations in anthropometric measures.

Most of the body composition in both human and rat is contributed by lean mass compared to fat mass [34,35]. In this study, despite an increase in fat mass, no significant changes in body weight among HCHF-induced rats were observed. In contrast, the C group showed an increase in body weight along with an increase in lean mass. The difference in the outcomes may have been due to differences in the protein composition of the diet. The rat chow diet contained a higher protein content between 21 and 23%, whereas the HCHF diet contained only 3.93–4.24% protein. Protein is important for muscle growth, contributing to an increase in lean mass [36]. A previous study showed that HCHF-induced rats had significantly lower body weight collateral with lower lean mass compared to chow-fed rats [22]. Therefore, an increase in lean mass simultaneously increases body weight.

The HCHF diet induction led to fat cell accumulation in muscle tissue and the degeneration of muscle fibers in rats, as observed through histomorphometric analysis [37]. Excessive fat intake results in the infiltration of fat into the muscles (intramuscular myosteatosis), hindering muscle growth and contributing to the progression of sarcopenic obesity [38]. Sarcopenic obesity is characterized by a decrease in skeletal muscle mass accompanied by an increase in fat mass [39]. As a result, individuals with sarcopenic obesity may exhibit normal body weight, BMI and waist circumference [40,41]. While sarcopenia typically affects the elderly, obesity accelerates its onset. Moreover, previous studies have shown that inflammation can activate muscle protein degradation, causing skeletal muscle atrophy [42,43]. Therefore, the body weight, BMI and abdominal circumference that did not increase in this study could be have been due to muscle degradation in sarcopenic obesity or inflammation that may have occurred in the HCHF-induced MS rats. This was further supported by a decrease in lean mass and an increase in TNF-α observed in HCHF-induced rats in this study.

The HCHF diet induction in this study caused increases in blood pressure. Histomorphometry findings in the kidney tissue showed an increase in Bowman’s space area and glomerular atrophy. The hypertension in this study can be attributed to multiple factors. Excess sodium and fructose can increase blood pressure by activating the renin–angiotensin–aldosterone system (RAAS) [44,45]. Excess adipose tissue can increase angiotensinogen secretion [46]. High leptin levels stimulate the sympathetic nervous system, which increases sodium reabsorption in the kidney [47,48]. Inflammatory marker TNF-α can stimulate the production of angiotensinogen and increase the production of reactive oxygen species (ROS) and decreasenitric oxide (NO), which acts as a vasodilator on the vascular endothelium [48,49]. Therefore, high fructose and high sodium in the HCHF diet, increased fat mass, high serum leptin and high TNF-α, which are also observed in this study, may contribute to the elevated blood pressure in the HCHF-induced rats.

Development of insulin resistance begins at the tissue level, primarily in adipose and skeletal muscle tissues, before progressing to hepatic tissue and eventually leading to systemic insulin resistance characterized by hyperinsulinemia [50]. Insulin resistance induced by the HCHF diet for 16 weeks may still be at its early stage, as suggested by previous studies [21,22]. This study further confirms the previous findings, as no systemic insulin resistance in this study occurred, as evidenced by no changes in fasting blood glucose, AUC OGTT, serum insulin and HOMA-IR. However, the study found that HCHF induced insulin resistance at the adipose tissue level, indicated by an increased L/A ratio [51]. Previous studies have shown that the HCHF diet induction for 18–20 weeks could induce insulin resistance in rats, as evidenced by increased HOMA-IR [52,53,54]. Therefore, it is likely that extending the period of HCHF diet induction beyond 16 weeks is necessary in this study to cause insulin resistance.

Insulin resistance is the key contributor to metabolic dyslipidemia [55]. Hepatic insulin resistance causes the failure of insulin-mediated secretion of very low-density lipoprotein (VLDL). Consequently, VLDL-rich TG increases in the blood and stimulates its conversion to LDL-rich TG and HDL-rich TG [56]. Subsequently, both are broken down by lipase enzymes into sd-LDL and sd-HDL. Sd-HDL is removed through renal clearance, reducing the HDL level [4]. However, the HCHF diet in this study increased serum TG but did not significantly alter serum TC, LDL and HDL compared to the control group. The elevation in serum TG could be attributed to the excessive intake of fructose and saturated fatty acid (SFA) in the HCHF diet, which can increase TG level in the bloodstream [57]. The lack of significant change in serum TC of the HCHF-induced rats compared to the C group could be due to the rat’s natural resistance to high blood cholesterol [22,58]. Meanwhile, hepatic insulin resistance in this study might not have fully developed, which could explain the absence of significant changes in serum LDL and HDL.

Excess free fatty acids (FFA) in obesity are known to play a significant role in stimulating inflammation [59]. The inflammation, often referred to as metabolic inflammation, is characterized by chronic low-grade inflammation that occurs locally within organs [60]. A previous study by Hashim et al. (2023) [21] reported that HCHF diet induction for 16 weeks did not increase TNF-α and IL-1β levels in rat’s serum. However, this study demonstrated an increase in TNF-α in adipose tissue, although no significant changes in IL-6 and IL-1β levels were observed. These findings suggest that the inflammation in MS induced by the HCHF diet is indeed a chronic low-grade inflammation detectable locally at the tissue level. Previous research has demonstrated that HCHF induction using animal fat tallow and lard oil as a source of fat could increase TNF-α, as well as IL-6 and IL-1β in adipose and liver tissue [53,61]. Therefore, substituting pure ghee with another source of fat could potentially increase all the inflammatory markers measured in this study.

Leptin levels positively correlate with increased body fat, whereas increased body fat is not correlated with serum adiponectin level [62]. This study demonstrated that the increase in fat mass was consistent with elevated serum leptin, with no significant changes in serum adiponectin and PPARγ levels in adipose tissue. PPARγ plays a crucial role in stimulating adiponectin secretion from adipose tissue [63,64]. Similar findings by a previous study that showed HCHF diet induction for 16 weeks increased serum leptin without changes in serum adiponectin [21]. Therefore, an increase in body fat will not necessarily have a suppressing effect on adiponectin levels. A previous study has revealed no correlation between serum adiponectin and body fat mass [62]. Mice fed with a high-fat diet for 30 weeks showed no changes in adiponectin expression in visceral adipose tissue and only exhibited decreased adiponectin expression in subcutaneous adipose tissue [65]. Furthermore, a human study found that metabolically obese women had lower adiponectin mRNA expression in subcutaneous compared to visceral adipose tissue [66]. These findings agree with the current study.

The 11βHSD1 enzyme plays an important role in activating corticosterone hormone [67]. Researchers have hypothesized that high corticosterone is likely to be one of the mechanisms involved in the formation of MS because both share the same metabolic effects such as obesity, hyperglycemia, dyslipidemia and hypertension [68,69]. However, HCHF induction for 16 weeks in this study could not demonstrate changes in serum corticosterone and 11βHSD1 in the adipose tissue in HCHF-induced rats. This agrees with the previous work by Hashim et al. (2023) [21]. Previous studies have shown that feeding mice a high-fat diet with 42–60% fat content could increase the expression of 11βHSD1 and plasma corticosterone [70,71,72]. However, the HCHF diet in the current study comprised 24.62% fat, which was significantly lower compared to previous studies. Hence, it is likely that a higher fat composition in the HCHF diet could increase corticosterone and 11βHSD1 in this study.

Treatment of DeGT and RGT in HCHF-induced rats reduced energy intake, which was consistent with a decrease in serum corticosterone observed in this study. It is well established that high corticosterone levels can increase the secretion of ghrelin, a hormone known to stimulate appetite [73,74]. Therefore, our findings suggest the treatment of DeGT and RGT reduces energy intake by lowering corticosterone levels, thereby potentially reducing ghrelin secretion.

DeGT and RGT treatments prevented obesity in rats in this study, as evidenced by a reduction in fat mass and adipocyte hypertrophy in histomorphometry findings. These findings align with the reduction in serum corticosterone and 11βHSD1 of adipose tissue in this study. It is known that corticosterone promotes fat accumulation by increasing adipogenesis [75]. A previous study also demonstrated raw SBH from *H. itama* species 1 g/kg could lower serum corticosterone and 11βHSD1 in adipose tissue [21]. However, only RGT treatment effectively reduced ectopic fat accumulation in liver tissue, as indicated by a reduction in hepatosteatosis grade.

This study demonstrated that both DeGT and RGT exerted a similar effect in lowering systolic and diastolic blood pressure. Histomorphometry of kidney tissue revealed a reduction in Bowman’s space area and glomerular atrophy in both treatment groups. This effect was likely attributed to the reduction of fat mass and TNF-α in adipose tissue observed in both treatment groups. Additionally, RGT may reduce hypertension due to a reduction in serum leptin. Previous studies have shown that raw SBH from *H. itama* species 1 g/kg could lower blood pressure [21,22,76].

However, DeGT treatment could not lower serum TG levels, unlike RGT in this study. This discrepancy could have been due to the dehydration process that has been shown to reduce the phenolic content in honey [77]. Heat processing can also reduce the bioactivity of flavonoids [78]. Previous research has shown that the *Solanum nigrum* extract, which contains phenolic and flavonoid, could decrease fatty acid synthase (FASN) and sterol regulatory element binding protein 1 (SREBP-1 activity) activities, both of which are involved in TG synthesis [79]. Another study showed that the litchi flower water extract rich in phenolic and flavonoid could inhibit pancreatic enzyme lipase activity involved in postprandial TG absorption and increase the removal of triacylglycerol fat through feces [80]. Therefore, the reduction of TG by RGT was likely due to its phenolic and flavonoid compounds, which may have been lost in DeGT due to the dehydration process.

Similarly, DeGT treatment could increase HDL, while RGT did not exhibit the same effect in this study. The dehydration process in this study may enhance the bioactive compounds in DeGT, as dehydration can activate new compounds or convert the existing compounds into a more stable form [81]. A previous study showed that luteolin, a flavonoid, increased the expression of liver X receptor (LXR) and ATP binding cassette transporter G1 (ABCG1), both of which helped in the HDL maturation process [82]. Therefore, the increase in HDL by DeGT in this study was likely due to bioactive content, which may have been enhanced following the dehydration process.

This study also found that both DeGT and RGT treatments reduced inflammation, as evidenced by the reduction of TNF-α in adipose tissue. Previous studies also demonstrated the local anti-inflammatory effect of RGT 1 and 2 g/kg, as well as dried SBH from *H. itama* species in the form of bee bread 0.5 g/kg, in reducing TNF-α levels in pancreas and liver, respectively [23,83]. The reduction of inflammation in this study was likely due to the decrease in fat mass observed in both treatment groups. Excess fat could activate the adipose tissue nuclear factor-kappa B (NF-κβ) and c-Jun N-terminal kinase (JNK) pathways that initiate inflammation [84]. Previous studies have shown that RGT and bee bread from *H. itama* species decreased TNF-α along with the decrease of NF-κβ [23,83]. However, this study showed that DeGT treatment could not lower IL-6 and IL-1β in adipose tissue, unlike RGT. This could have been due to the absence of bioactive compounds in DeGT that could lower inflammatory cytokines IL-6 and IL-1β.

**Figure 12:**
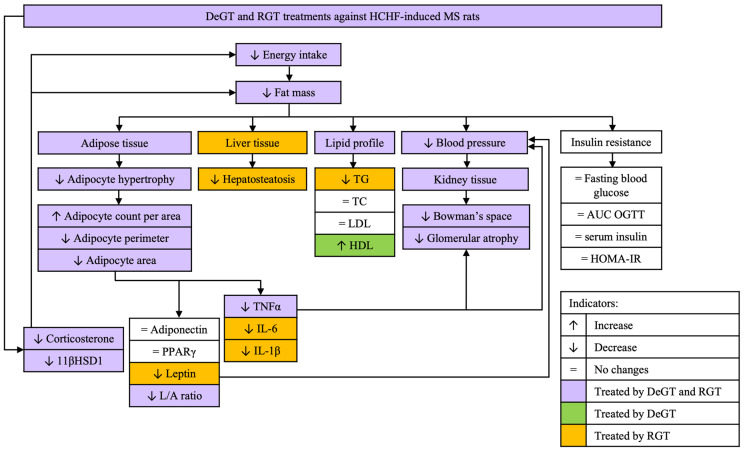
Summary of the effect of DeGT and RGT treatments against HCHF-induced MS.

DeGT treatment also could not lower serum leptin, unlike RGT. However, both DeGT and RGT treatments effectively reduced the serum L/A ratio. This suggests that although DeGT did not directly impact the serum leptin level, DeGT decreased the leptin ratio per adiponectin level in rats’ serum. Moreover, the L/A ratio indicates insulin resistance in adipose tissue because it accurately measures adipose tissue dysfunction [51,85]. A previous study showed that rapid and excessive adipocyte expansion caused cell hypoxia, leading to insulin resistance in adipose tissue [86]. The ability of both DeGT and RGT treatments to reduce the L/A ratio was consistent with the reduction of adipocyte hypertrophy as shown by both treatment groups, suggesting their potential in mitigating insulin resistance in adipose tissue. Figure 12 summarizes the effect of DeGT and RGT treatments observed in this study.

Honey with moisture content below 17% can prevent fermentation by microorganisms [25,87]. Therefore, a dehydration process is necessary for SBH to reduce its moisture content. However, high-heat treatment can increase the HMF content in honey [88]. Excessive HMF is harmful to health due to its potential to act as a carcinogenic and genotoxic agent [89,90]. Moreover, heat treatment can cause denaturation of the diastase enzyme and a reduction in its activity [91]. Meanwhile, ash content reflects the mineral content in honey [92]. In this study, the dehydration process reduced DeGT moisture content below 17%, reduced its acidity, caused no increase in HMF and maintained diastase enzyme activity and ash content. However, there were decreased trehalulose, fructose and glucose in DeGT, while the content of sucrose and maltose remained unchanged. A previous study showed that the increased heat treatment on honey decreased its glucose content, likely due to heat causing the degradation of sugar content [93].

DeGT can treat certain MS parameters as effectively as RGT in this study, possibly due to their similar bioactive compounds. Among them are trehalulose, glutamine and epigallocatechin gallate (EGCG). Previous studies have demonstrated that these compounds have potential benefits in treating various components of MS. Trehalose can effectively treat adipocyte hypertrophy [19]. Meanwhile, glutamine can reduce body fat mass, lower high blood pressure and increase lean body mass [94]. EGCG can reduce body fat mass, serum corticosterone, 11βHSD1 in adipose tissue, high blood pressure and enlargement of Bowman’s space [21,67,95].

However, DeGT was unable to treat some MS parameters like RGT, possibly due to the differences in its phenolic content. The dehydration process can reduce the phenolic content in honey [77]. Moreover, heat treatment can also reduce the bioactivity of flavonoids [78]. In this study, RGT contained sinapic acid, which was not found in DeGT. Previous studies have shown that sinapic acid can lower serum TG, leptin, IL-6 and hepatosteatosis [96,97,98,99].

DeGT can treat certain MS parameters better than RGT, possibly due to the activation of several new compounds. A previous study has shown dehydration can activate new compounds and convert existing compounds into a more stable form [81]. In this study, DeGT contained raffinose, luteolin, Rehmannioside A and quinic acid derivatives, which were not found in RGT. These compounds have been shown by previous studies to increase serum HDL [82,100,101,102].

To the best of our knowledge, this is the first study that compares the potential of dehydrated and raw SBH in MS treatment. The dehydration process in this study involves heat exposure, which could potentially reduce phenolic content or flavonoid bioactivity in DeGT. This factor may have caused DeGT to be less effective in treating some MS components than RGT. Using the freeze-drying method as a dehydration process could prevent honey from being exposed to heat. This may increase the probability of DeGT’s potential to treat MS equally or more effectively than RGT.

## 4. Materials and Methods

### 4.1. Animals

The sample size was calculated using resource equation approach as explained by Arifin and Zahiruddin (2017) based on the following formula: *n* = DF/*k* + 1 (*n* = number of animals per group, DF = degree of freedom, *k* = number of groups) [103]. The DF in the formula was replaced with the minimum (10) and maximum (20) to obtain the minimum and maximum numbers of animals per group, respectively. The minimum *n* = 10/*k* + 1 = 10/4 + 1 = 3.5, and the maximum *n* = 20/*k* + 1 = 20/4 + 1 = 6. Hence, the total minimum and maximum numbers of animals (*N*) required were: minimum *N* = minimum *n* × *k =* 3.5 × 4 = 14, and maximum *N* = maximum *n* × *k* = 6 × 4 = 24. The maximum number of rats were adopted. Therefore, total *N* = 24 rats, with *n* = 6 rats per group.

This study has received approval from the Animal Ethics Committee, Universiti Kebangsaan Malaysia, with approval code ANAT/FP/2021/FAIRUS/24-NOV./1210-JAN.-2022-SEPT-2023. Twenty-four male Wistar rats weighing 250–300 g were obtained from the Laboratory Animal Resource Unit, Faculty of Medicine, Universiti Kebangsaan Malaysia. The rats were caged individually and acclimatized for two weeks in the Animal Laboratory Department of Anatomy, Faculty of Medicine, Universiti Kebangsaan Malaysia. The laboratory condition was standardized at 12 h of light/dark cycle and an ambient temperature of 25 ± 3 °C.

### 4.2. HCHF Diet Preparation

HCHF diet contains mixture of 155 g rat chow powder (Gold Coin Feedmills (M), Port Klang, Malaysia); 175 g D-(−)-Fructose fructose powder (Chemiz, Shah Alam, Malaysia); 395 g sweetened condensed milk (Gold Coin, F & N Diaries Manufacturing Sdn. Bhd., Malaysia); 200 g pure ghee (Enrico’s Pure Ghee, RaviRaj Sdn. Bhd., Pulau Pinang, Malaysia); 25 g mixture of Hubble, Mendel and Wakeman salts (MP Biomedicals, Santa Ana, CA, USA); and 50 g tap water, as well as 25% fructose in drinking water [45].

### 4.3. Dehydrated and Raw SBH Preparation

Raw SBH from *G. thoracica* species (RGT) was harvested in April 2022 from a bee farm in Gombak, Selangor, Malaysia. Immediately after harvesting, 1 kg of the RGT underwent dehydration at 32–39 °C for 20 h. The dehydration process of the dehydrated SBH (DeGT) was performed using Malaysian Agricultural Research and Development Institute (MARDI) dehydration machine [104]. The treatment dosage used in this study was 1.0 g/kg/day for RGT [22] and 0.833 g/kg/day for DeGT. The calculation of the DeGT treatment dose is shown in Table 3. Both DeGT and RGT were diluted with distilled water at a 1:1 ratio during administration to rats.

### 4.4. Honey and LC-MS Analysis

The physicochemical properties analysis of honey was conducted by UNIPEQ Sdn. Bhd. (UKM-MTDC Technology Center, Bangi, Malaysia) using the following equipment: moisture content, ATAGO^®^ pocket refractometer (ATAGO Co., Ltd., Tokyo, Japan); pH, Sartorius PB-10 pH meter (Sartorius, Göttingen, Germany); free acidity, titration and Sartorius PB-10 pH meter (Sartorius, Germany); HMF, Waters e2695 UV Detector HPLC machine (Waters, Milford, MA, USA); ash content, carbolite furnace chamber machine (Carbolite Gero Ltd., Hope Valley, UK); diastase activity, Thermo Scientific Helios Zeta UV-Vis spectrophotometer (Thermo Fisher Scientific Inc., Waltham, MA, USA); and sugar content (trehalulose, fructose, glucose, sucrose, maltose), high-performance liquid chromatography (HPLC) Varian 385-LC ELSD machine (Varian, Inc., Palo Alto, CA, USA).

LC-MS analysis was conducted by Advanced Chemistry Solutions Company (Petaling Jaya, Selangor, Malaysia) using an ultra-high-performance liquid chromatography (UHPLC) machine, ACQUITY UPLC I-Class system (Waters, USA) and Vion IMS QTOF hybrid mass spectrometer (Waters, USA).

### 4.5. Study Design

The 24 male Wistar rats were assigned randomly into four groups (*n* = 6 for each group): control (C), HCHF-induced MS without treatment (MS), HCHF-induced MS treated with DeGT (MS+DeGT) and HCHF-induced MS treated with RGT (MS+RGT). Group C received rat chow, while the other groups were fed with HCHF diet ad libitum for 16 weeks. The initial eight weeks of HCHF induction aimed to ensure the successful development of MS by measuring relevant parameters before treatment. In the subsequent eight weeks (from week 9 to week 16), two groups of HCHF diet-induced MS rats (MS+DeGT and MS+RGT) received their respective supplements via oral gavage. The nutritional compositions of the rat chow [105] and HCHF diets are included in Table S1 and Table S2, respectively.

Food and drink intake for each rat were measured daily. MS parameters were taken at baseline, week 8 and week 16. The parameters for obesity were assessed by anthropometric measurements (body weight, BMI and abdominal circumference) and body composition (fat mass and lean mass). The hypertension parameter was assessed by blood pressure measurement (systolic and diastolic). The insulin resistance parameter was assessed by measurement of fasting blood glucose, OGTT, serum insulin and HOMA-IR. The levels of TG and HDL were assessed by serum fasting lipid profile (comprised of serum TG, TC, LDL and HDL). MS is diagnosed when at least three out of five key risk factors are present [21,22]. The presence of a condition was defined as a significant difference for the indices between the rats fed with HCHF diet compared to those fed with standard rat chow.

At week 16, all rats were euthanized using intravenous injection of Dorminal 20% (contains 200 mg/mL pentobarbital sodium) at a dosage of 0.7 mL/kg body weight. Serum and visceral adipose tissue were collected for enzyme-linked immunosorbent assay (ELISA) analysis to measure inflammatory and obesity markers. Organs such as visceral adipose tissue, liver and kidney were harvested for histological analysis.

### 4.6. Energy Intake Measurement

Energy values were calculated according to a previous study by Wong et al. (2018) [45] as follows: energy intake (kJ/day) = [food intake (g) × energy value (kJ/g)] + [drink intake (mL) × energy value (kJ/mL)]. Daily energy intakes from week 1 until week 8 were averaged (Week 1–8), and daily energy intakes from week 9 until week 16 were averaged (Week 9–16).

### 4.7. Anthropometric Measurement

Body weight was measured using a Nimbus^®^ balance scale (Adam Equipment, Inc., Oxford, CT, USA). Abdominal circumference was measured at the widest area of the abdomen, and the rat’s length was measured from the tip of the nose to the anus using a non-stretch plastic measuring tape (Birch Creative, Heidelberg West, VIC, Australia). BMI was calculated using the formula: BMI (g/cm^2^) = weight (g)/length (cm^2^).

### 4.8. Body Composition Measurement

Body composition, including fat and lean mass, was measured using DXA scan Hologic Discovery W and Small Animal Analysis Software 13.5.3, Hologic QDR-1000 System (Hologic Inc., Marlborough, MA, USA). Rats were anaesthetized prior DXA scan by intravenous injection of a ketamine–xylazine–Zoletil mixture at 0.1 mL/100 g of body weight.

### 4.9. Blood Pressure Measurement

Before the measurements, rats were placed in a holder tube and kept on a heating pad to maintain the rat’s tail temperature at 33–35 °C. Systolic and diastolic blood pressure were then measured using a CODA^®^ non-invasive blood pressure system machine (Kent Scientific Corporation, Torrington, CT, USA).

### 4.10. Fasting Blood Glucose and OGTT Measurement

All rats were fasted for 12 h and provided only with tap water. Prior to OGTT, minimal bleeding was done at the rat’s tail. Fasting blood glucose was measured using an Accu-Chek^®^ Instant S glucometer (Roche Diagnostics, Basel, Switzerland). Subsequently, a 40% glucose solution (D(+)-glucose, Chemiz, Shah Alam, Malaysia) at a dosage of 2.0 g/kg body weight was given to the rats via oral gavage. Blood glucose readings were then recorded at 30, 60, 90 and 120 min after glucose loading. The AUC of OGTT was calculated using the trapezoidal rule.

### 4.11. Serum Insulin and HOMA-IR Measurement

Before blood sampling, all rats were fasted for 12 h. Anesthesia was induced by intravenous injection of a ketamine–xylazine–Zoletil mixture at 0.1 mL/100 g body weight. Retro-orbital bleeding was performed, and blood samples were centrifuged to isolate serum using Thermo Scientific Heraeus Biofuge Primo R (Thermo Fisher Scientific Inc., USA) at 4000 rpm and 20 °C for 15 min. Serum insulin was determined using an ELISA kit (Finetest^®^, Wuhan, China). HOMA-IR was calculated using the formula, HOMA-IR = fasting blood glucose (mmol/L) × fasting insulin (µU/mL)/22.5 [106].

### 4.12. Serum Fasting Lipid Profile Measurement

The serum fasting lipid profile (TG, TC, LDL and HDL) was analyzed at the Veterinary Laboratory Services Unit (VLSU), Faculty of Veterinary Medicine, Universiti Putra Malaysia (UPM), Serdang, Malaysia using integrated chemistry system Dimension^®^ Xpand^®^ Plus (Siemens, Munich, Germany).

### 4.13. Determination of Inflammatory and Obesity Markers Using ELISA

Samples for the determination of inflammatory markers were adipose tissue homogenate, while samples for the determination of obesity markers were serum and adipose tissue homogenate. Adipose tissue homogenization was performed employing the procedures outlined in the previous study by Hashim et al. (2023) [21]. The inflammatory markers (TNF-α, IL-6 and IL-1β) and obesity markers (leptin, adiponectin and PPARγ), including corticosteroid synthesis (corticosterone and 11βHSD1), were determined using the ELISA technique (Finetest^®^, China). The L/A ratio was calculated by dividing the serum leptin levels (ng/mL) by serum adiponectin levels (ng/mL) [51]. Protein content in adipose was quantified using a bicinchoninic acid (BCA) protein colorimetric assay kit (Elabscience^®^, Houston, TX, USA).

### 4.14. Histomorphometry Examination and Analysis

Adipose tissue, liver and kidney were fixated in 10% formaldehyde. All tissues were processed, embedded, sectioned at 5 μm thickness, stained with hematoxylin and eosin using Autostainer XL (Leica, Wetzlar, Germany) and mounted on a glass slide. Histomorphometric examination was conducted at 100× and 400× total magnification using a Carl Zeiss Primo Star HD light microscope equipped with Zeiss ZEN Microscopy Imaging System software version 2.6 (Zeiss, Oberkochen, Germany). Histology analysis was performed using ImageJ software 1.54 (National Institutes of Health, Bethesda, MD, USA) as shown in Table 4.

### 4.15. Statistical Analysis

All data were analyzed using the Statistical Package for the Social Science (SPSS^®^) version 26 software (IBM, Armonk, NY, USA), with statistical significance defined at *p*-value < 0.05. The normality of data distribution was assessed using the Shapiro–Wilk test. Normally distributed data were analyzed using one-way ANOVA with Tukey’s post-hoc comparison. Data with a time × group design were analyzed using mixed design analysis of variance (ANOVA) with a small effect analysis as the post hoc test. Non-normally distributed data were analyzed using Kruskal–Wallis test.

## 5. Conclusions

DeGT is as effective as RGT in treating various MS-related factors, including energy intake, fat mass, hypertension, serum L/A ratio, serum corticosterone and 11βHSD1 in adipose tissue, TNF-α in adipose tissue, adipocyte hypertrophy, enlargement of Bowman’s space area and glomerular atrophy. DeGT increased HDL levels, but DeGT was unable to reduce serum TG, IL-6 and IL-1β in adipose tissue, serum leptin and hepatosteatosis compared to RGT. In conclusion, DeGT demonstrated its potential benefits as a preventive agent for MS, but RGT showed better anti-MS agents than DeGT in this study. Prolonging the duration of HCHF diet induction or increasing its fat composition could potentially induce more MS risk factors. Meanwhile, extending the duration of DeGT treatment may increase its efficacy in treating MS. To enhance the potential of DeGT in treating MS, future research can explore alternative dehydration methods like freeze-drying, which avoids heat exposure and may yield better results than the current dehydration process. Considering the promising results in this study, we hope to explore the potential benefits in humans in the future, given that SBH is a natural product proven to be effective in preventing MS in animal studies.

## Figures and Tables

**Table 1:** Physicochemical properties in DeGT and RGT.

Physicochemical Properties	Unit	DeGT	RGT
Moisture content	%	14.3	31
pH		3.5	3.3
Free acidity	mEq/kg	138	274.8
Hydroxymethylfurfural (HMF)	mg/kg	<0.1	0.3
Ash content	g/100 g	0.2	0.2
Diastase activity	DN	<1	<1
Trehalulose	g/100 g	48.5	52
Fructose	g/100 g	8.1	8.5
Glucose	g/100 g	7.3	11
Sucrose	g/100 g	<0.01	<0.01
Maltose	g/100 g	<0.01	<0.01

**Table 2:** Bioactive compounds in DeGT and RGT.

Bioactive Compounds	DeGT	RGT
Sugar	Isomaltose, mannotriose, stachyose, meso-inositol, fuzinoside	
	Raffinose, galactose, α-kojibiose, 1F-Fructofuranosylnystose, Helonioside B	2′,6′-Diacetyl-3,6-diferuloylsucrose
Amino acid	Glutamine, tyrosine	
Phenolic	Calycanthoside, dihydro-3-coumaric acid	
	-	Sinapic acid
Flavonoid	Epigallocatechin(4β,8)-gallocatechin, 6″-O-p-Hydroxybenzoyliridin, Glehlinoside B	
	Luteolin-7-O-α-D-glucoside	5′-Methoxy-bilobetin
Iridoid glycoside	Sesamoside, Geniposidic acid, Secoxyloganin	
	Rehmannioside A, Shanzhiside methyl ester	-
Phenylpropanoids	Phenylpropionic acid	
Other organic compounds	Cnideoside A, Manhuannin J, Methyl 3,4-dihydroxy-phenyllactate, 1,3,6-Trihydroxy-2-methylanthraquinone-3-O-(O-6-acetyl) neohesperidin,	
	5-Hydroxymethyl furoic acid, 1-O-Methyl-3,5-O-dicaffeoylquinic acid methyl ester, Trimethyl citrate, Meliadanoside B, 2-Hydroxy-5-methoxy acetophenone	Mudanpioside H, Sibiricaxanthone B
Total	33 bioactive compounds	25 bioactive compounds

**Table 3:** Calculation of DeGT treatment dose.

	Calculation
Moisture content	Moisture content RGT = 31%, moisture content DeGT = 14.3%. (based on honey analysis in Table 1)
Moisture reduction	Moisture reduction = moisture content RGT − moisture content DeGT ∴ Moisture reduction=31%−14.3%=16.7%
Treatment dose DeGT	Treatment dose of RGT = 1.0 g/kg/day [22] Treatment dose DeGT = treatment dose RGT − (moisture reduction × treatment dose RGT) $∴\;\mathrm{Treatment}\;\mathrm{dose}\;\mathrm{DeGT}=1.0-\left(\frac{16.7}{100}\times 1.0\right)=0.833\;g/kg/day$

**Table 4:** Parameters measured for histological analysis.

	Parameters
Adipose tissue	Adipocyte count, area and perimeter [22].
Liver tissue	Hepatosteatosis grading was based on the percentage of fat in liver cells [107]: Grade 0 = <5%, grade 1 = 5–33%, grade 2 = 34–66% and grade 3 = >66%
Kidney tissue	Bowman’s space area calculation [108]: Area of Bowman’s space = area of renal corpuscle–area of glomerulus

## Data Availability

The original contributions presented in the study are included in the article/Supplementary Material, further inquiries can be directed to the corresponding author.

## Supplementary Materials

The following supporting information can be downloaded at: https://www.mdpi.com/article/10.3390/ph17111427/s1, Table S1: Nutritional composition of rat chow diet (Gold Coin Feedmills (M), Malaysia); Table S2: Nutritional composition of HCHF diet.
