# Supplementary material for: The Potential of Dehydrated Geniotrigona thoracica Stingless Bee Honey against Metabolic Syndrome in Rats Induced by a High-Carbohydrate, High-Fat Diet
Source: Pharmaceuticals (Basel). 2024 Oct 24;17(11):1427. doi: 10.3390/ph17111427 (PMC11597213; doi:10.3390/ph17111427)
Supplement: Supplementary file 1 [file pharmaceuticals-17-01427-s001.zip › pharmaceuticals-3265448-supplementary.pdf]

Table S1. Nutritional composition of rat chow diet (Gold Coin Feedmills (M), Malaysia).

Composition	Amount/percentage
Crude protein	21 – 23%
Crude fibre (max)	5.0%
Crude fat (min)	3.0%
Moisture (max)	3.0%
Calcium	0.8 – 1.2%
Phosphorus	0.6 – 1.0%
Nitrogen free extract	49.0%
Vitamin A	10 M.I.U.
Vitamin D_3	2.5 M.I.U.
Vitamin E	15 g
Vitamin K	trace
Vitamin B_{12}	trace
Thiamine	trace
Riboflavin	trace
Pantothenic acid	trace
Niacin	trace
Pyridoxine	trace
Choline	trace
Santoquin	trace
Microminerals	trace

Table S2. Nutritional composition of HCHF diet.

Composition	Amount/percentage in HCHF diet components	Total amount/percentage
Carbohydrate	Sweetened condensed milk = 23.62%<br>Fructose powder = 17.50%<br>Pure ghee = 0%	41.12%
Fat	Pure ghee = 19.96%<br>Sweetened condensed milk = 4.19%<br>Powdered rat chow = 0.47%	24.62%
Protein	Powdered rat chow = 3.26 – 3.57%<br>Sweetened condensed milk = 0.67%<br>Pure ghee = 0.02%	3.93 – 4.24%
Moisture	Water = 5.0%<br>Powdered rat chow = 0.47%<br>Pure ghee = 0.07%	5.54%
Fibre	Powdered rat chow = 0.78%	0.78%
Sodium	Hubble, Mendel and Wakeman salt mixture = 0.18%<br>Sweetened condensed milk = 0.07%<br>Pure ghee = trace	0.25%
Calcium	Powdered rat chow = 0.12 – 0.19%<br>Pure ghee = 0.01%	0.13 – 0.20%
Phosphorus	Powdered rat chow = 0.09 – 0.16%<br>Pure ghee = trace	0.09 – 0.16%
Cholesterol	Sweetened condensed milk = 0%<br>Pure ghee = 0%	0%
Others	<u>Powdered rat chow</u><br>Nitrogen free extract	7.60%
	<u>Hubble, Mendel and Wakeman salt mixture</u><br>Calcium carbonate = 1.36%<br>Magnesium carbonate = 0.06%<br>Magnesium sulfate.7H2O = 0.04%<br>Potassium chloride = 0.28%<br>Potassium phosphate monobasic = 0.53%<br>Ferric phosphate = 0.05%<br>Potassium iodide, Manganese sulfate.H2O, Aluminum potassium sulfate, Copper sulfate.5H2O = trace	2.32%
	<u>Pure ghee</u><br>Iron, zinc, magnesium	trace

Nutritional composition based on 155 g rat chow powder (Gold Coin Feedmills (M), Malaysia), 175 g D-(-)-Fructose fructose powder (Chemiz, Malaysia), 395 g sweetened condensed milk (Gold Coin, F&N Diaries Manufacturing Sdn. Bhd., Malaysia), 200 g pure ghee (Enrico's Pure Ghee, RaviRaj Sdn. Bhd., Malaysia) and 25 g mixture of Hubble, Mendel and Wakeman salts (MP Biomedicals, USA).
